# Spatiotemporal Diffusion, Colonization, and Antibody Responses in Susceptible C57BL/6J Mice Orally Infected with *Toxoplasma gondii* Cysts

**DOI:** 10.3390/vetsci12030212

**Published:** 2025-03-01

**Authors:** Zhao Li, Qi-Shuai Liu, Jun-Jie Hu, Cai-Qin Deng, Tao Li, Wen-Bin Zheng, Xing-Quan Zhu, Feng-Cai Zou

**Affiliations:** 1 Faculty of Animal Science and Technology, Yunnan Agricultural University, Kunming 650201, China; lizhao@ynu.edu.cn; 2 Animal Research and Resource Center, School of Life Sciences, Yunnan University, Kunming 650500, China; liuqishuai@ynu.edu.cn (Q.-S.L.); dengcaiqin1998@163.com (C.-Q.D.); 20224024@ynu.edu.cn (T.L.); 3 School of Ecology and Environmental Science, Yunnan University, Kunming 650500, China; jjhu@ynu.edu.cn; 4 Laboratory of Parasitic Diseases, College of Veterinary Medicine, Shanxi Agricultural University, Jinzhong 030801, China; wenbinzheng1@126.com; 5 The Yunnan Key Laboratory of Veterinary Etiological Biology, College of Veterinary Medicine, Yunnan Agricultural University, Kunming 650201, China

**Keywords:** *Toxoplasma gondii*, C57BL/6J mice, spatiotemporal diffusion, colonization, predilection sites, antibody fluctuation

## Abstract

*Toxoplasma gondii* is an obligate intracellular protozoan that infects humans and other mammals. The C57BL/6J mouse strain is regarded as an ideal model organism for studying *T. gondii* due to its susceptibility to *T. gondii* infection and its other advantages over other laboratory animals. However, systematic studies on the response dynamics of the susceptible C57BL/6J mice after oral infection with *T. gondii* cysts are lacking. To address this research gap, we investigated the spatiotemporal dynamics of infection, colonization, and antibody fluctuations in susceptible C57BL/6J mice orally infected with Type II *T. gondii* ME49 strain cysts. Mice were orally challenged with *T. gondii* cysts to examine the infection dynamics. Daily monitoring was conducted for 60 days post-infection (dpi) to assess animals’ clinical signs and survival rates. The parasite burden in various organs was quantified using qPCR targeting the *T. gondii* B1 gene. The serum antibody responses were evaluated using ELISA. The cyst burden in the mouse brain was assessed via histology and immunofluorescence. *T. gondii* infection induced clinical symptoms in the mice, including fever and weight loss. *T. gondii* rapidly invaded the mice’s small intestine, spleen, lungs, liver, and heart via the bloodstream within 1–5 dpi. *T. gondii* had breached the blood–brain barrier and colonized the brain by 7 dpi. The levels of *Toxoplasma*-specific IgG antibodies increased and stabilized for two months (until the experiment ended). Systemic parasite dissemination occurred rapidly, infiltrating most tissues and organs, leading to pronounced enteritis and multi-organ damage due to inflammation. The tachyzoites differentiated into bradyzoites when *T. gondii* infection progressed from the acute to the chronic phase in mice, forming tissue cysts in organs, including the muscles and brain. As a result, the predilection site of *T. gondii* in mice is the brain, which is where the cysts persisted for the host’s lifetime and continuously induced meningitis. These findings provide valuable insights into the spatiotemporal diffusion, colonization, predilection sites, temporal antibody dynamics, pathogen detection methodologies, and histopathological changes in C57BL/6J mice following oral infection with *T. gondii* cysts. These insights are important for elucidating *T. gondii*’s pathogenesis and host–*T. gondii* interaction.

## Simple Summary

*Toxoplasma gondii* is an important zoonotic parasite infecting humans and the majority of other warm-blooded animals. The C57BL/6J mouse is susceptible to *T. gondii* infection and is considered an ideal model organism for *T. gondii* studies. This research explored the spatiotemporal dynamics of infection, colonization patterns, and antibody response fluctuations in C57BL/6J mice orally infected with *T. gondii* Type II strain cysts. The mice were orally infected with *T. gondii* cysts, and their clinical symptoms were monitored daily. The parasite load in various organs was assayed using qPCR targeting the *T. gondii* B1 gene. Serum antibody responses were assessed using ELISA. The cyst burden in the mouse brain was evaluated through histological analysis and immunofluorescence. *T. gondii* infection led to clinical manifestations in mice, such as fever and weight loss. The parasite rapidly invaded the animals’ small intestine, spleen, lungs, liver, and heart through the bloodstream and breached the blood–brain barrier to colonize the brain. The production of *Toxoplasma*-specific IgG antibodies increased and remained stable for two months (i.e., until the end of the experiment). The parasite disseminated rapidly throughout the body, infiltrating most tissues and organs and causing severe enteritis and multi-organ damage due to inflammation. Our findings offer valuable insights into spatiotemporal spread patterns, colonization, predilection sites of infection, dynamic antibody responses, and histopathological changes in C57BL/6J mice after oral infection with *T. gondii* cysts. These insights advance our understanding of the mechanisms underlying *T. gondii* pathogenesis and host–*T. gondii* interaction.

## 1. Introduction

*Toxoplasma gondii* is a single-celled zoonotic protozoan parasite that infects a wide range of warm-blooded animals, including humans, other mammals, and birds [1,2]. It is estimated to infect approximately one-third of the global population [3]. The parasite’s life cycle typically includes tachyzoite, bradyzoite, and oocyst stages, which vary across host types. In intermediate hosts such as humans, non-feline mammals, and birds, *T. gondii* reproduces asexually and forms tachyzoites during the acute phase of infection. When the infection progresses to a chronic stage, tachyzoites convert into bradyzoites, which persist within the host for its entire lifetime. In definitive hosts (e.g., cats and other felids), *T. gondii* undergoes sexual reproduction in the intestines, producing oocysts excreted in feces [4]. These oocysts are highly resistant to environmental conditions and can infect new hosts [5]. Additionally, definitive hosts may generate tachyzoites and bradyzoites. Dormant bradyzoites persist in host tissues, evade the immune system, and spread through the food chain to other intermediate and definitive hosts [6,7].

Humans acquire *T. gondii* infection primarily through the oral ingestion of undercooked meat containing cysts or by consuming food and water contaminated with oocysts. Mother-to-child transmission via the placenta and transmission via blood transfusion or organ transplant are also potential infection routes [8]. Infections among immunocompetent adults are often asymptomatic. Typical symptoms such as fever, fatigue, swollen lymph nodes, and muscle pain may manifest in some cases. Acute infections in healthy individuals usually resolve spontaneously, whereas immunocompromised individuals can experience severe complications, such as pneumonia and encephalitis [9]. This condition can also increase the risk of miscarriage or congenital malformations in pregnant women [10,11,12]. Additionally, acutely infected immunosuppressed patients (e.g., those with cancer or HIV/AIDS) are increasingly vulnerable to reactivation of *T. gondii* cysts, causing severe retinitis [13] and central nervous system inflammation [14]. *T. gondii* and toxoplasmosis constitute a concern for individual and public health, food safety, and ecological stability, emphasizing comprehensive research on prevention, control, and treatment [5].

The C57BL/6J mouse is considered an important model organism due to its stable genetic background, complete telomere-to-telomere reference genomes [15,16], advanced gene editing technologies [17], ease of breeding, and cost-effectiveness attributes [18]. The C57BL/6J mouse is susceptible to *T*. *gondii* infection and has many advantages over other laboratory animals. These mice have been commonly employed in research focusing on the virulence of *T. gondii*, the development of vaccines, interactions between pathogens and hosts, and food safety [19]. Despite these applications, systematic studies on the infection dynamics of the C57BL/6J mice after oral infection with *T. gondii* cysts remain lacking. Mice are conventionally categorized into outbred and inbred strains. Outbred strains include ICR (CD-1), KM, and NIH, while typical inbred strains are C57BL/6, BALB/c, C3H, DBA/2, FVB, and SJL [20]. KM mice are most widely used in China, whereas ICR mice are more popular internationally [21]. Studies have reported that different strains exhibit varying levels of susceptibility and pathogenicity to *T. gondii*. Inbred strains typically possess higher susceptibility than outbred strains [22]. Specifically, C57BL/6 mice are more susceptible than BALB/c mice and are more likely to develop severe, lethal enteritis [23,24]. Additionally, mice with the C57BL/KsJ (H2(d) haplotype) and CB10-H2 (H2(b) haplotype) backgrounds are particularly prone to congenital toxoplasmosis [25].

The infection stage (tachyzoites, cysts, and oocysts), contamination route (e.g., oral gavage, intraperitoneal injection, tail vein injection, and subcutaneous injection), and phase (acute or chronic) largely impact pathogenicity and immune responses in mice [26,27]. Further investigation is necessary to clarify the underlying mechanisms of pathogenesis and the dynamic immune responses in C57BL/6 mice infected via natural oral routes.

*T. gondii* is classified as a primary pathogen that should be excluded from specific pathogen-free laboratory mice. C57BL/6 mice are commonly used as the genetic background for genetically modified mice [17]. A *T. gondii* infection model in C57BL/6 mice is required to investigate immune responses and interactions during *T. gondii* infection.

This study explored the spatiotemporal diffusion, colonization patterns, and antibody response fluctuations in C57BL/6J mice infected orally with Type II *T. gondii* ME49 strain cysts [23]. These animals’ clinical signs, body weights, temperatures, and survival rates were monitored daily for 60 days post-infection (dpi). Parasite burden in various organs was quantified using qPCR targeting the *T. gondii* B1 gene. Serum antibody responses were assessed with enzyme-linked immunosorbent assay (ELISA). We also evaluated the brain cyst burden during chronic infection using histology and immunofluorescence.

## 2. Materials and Methods

### 2.1. Parasite Strains, Cell Culture Conditions, and Purification

The *T. gondii* strain TgME49 was maintained at 37 °C with 5% CO_2_ in confluent monolayers of human foreskin fibroblast cells following the method previously described [28]. Tachyzoites were harvested from highly infected human foreskin fibroblasts by mechanical release with a 27-gauge needle, followed by filtration through a 5 µm polycarbonate membrane filter to obtain pure parasites for subsequent mouse infection.

### 2.2. Mice and T. gondii Infection

**Figure 1:**
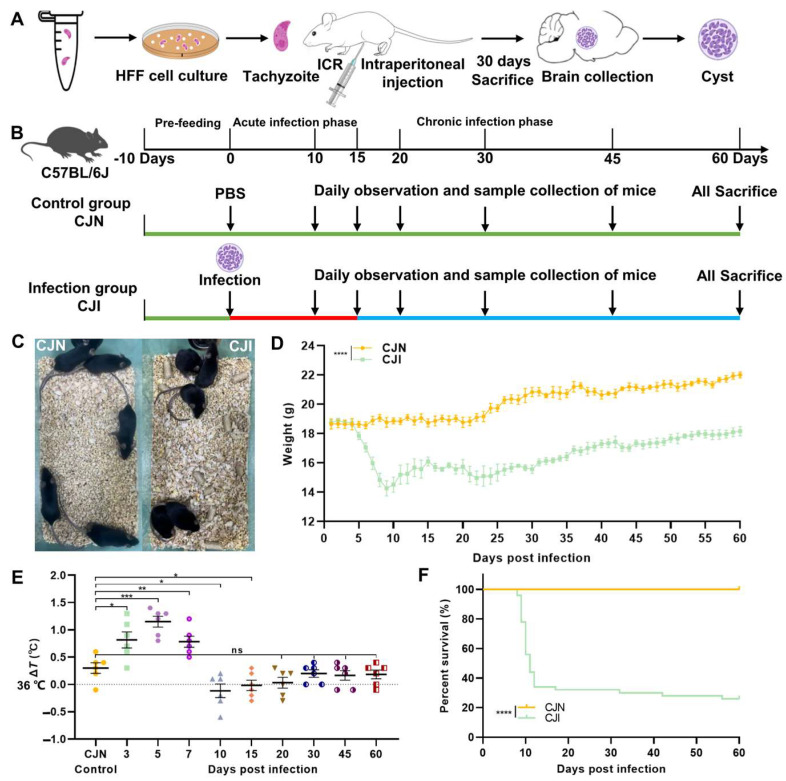
Establishment of C57BL/6J mouse model with *T. gondii* infection and observation of clinical symptoms. (**A**) *T. gondii* tachyzoites were cultured in vitro within host cells, and *T. gondii* cysts were cultured in mice. (**B**) Schematic diagram of experimental grouping and sampling for establishing *T. gondii* infection model in C57BL/6J mice. (**C**) Observations of daily behavior in CJN and CJI mice. (**D**) Body weight changes in C57BL/6J mice. Statistical significance was determined using two-way ANOVA. (**E**) Changes in rectal temperature with 36.0 °C as baseline to calculate body temperature changes. (**F**) Survival curves of C57BL/6J mice infected with cysts of TgME49 strains. C57BL/6J mice were infected by oral gavage using PBS and TgME49 cysts, and host survival was monitored for 60 days. Survival rate was calculated using log-rank (Mantel–Cox) test. Data are presented as mean ± SEM. All experimental data for control and treatment groups cover six biological replicates in each group. Statistical significance was determined using Student′s *t*-test. ^ns^ *p* > 0.05, * *p* < 0.05, ** *p* < 0.01 *** *p* < 0.001, and **** *p* < 0.0001.

Female ICR and C57BL/6J mice aged 6–8 weeks were raised under specific pathogen-free conditions at the Animal Research and Resource Center of Yunnan University (Approval Nos. SCXK-Yunnan-K2021-0001, SYXK-Yunnan-K2021-0002, and CNAS LA0029). The housing conditions conformed to the national standards: an environmental temperature of 22 ± 1 °C, relative humidity of 50 ± 5%, and 12 h light/dark cycle. Mice were provided with an irradiated and sterilized diet (JXT Standard) and had ad libitum access to filtered sterile water. Each ICR mouse was intraperitoneally injected with 500 *T. gondii* tachyzoites, and their weight changes, diet, and water intake were monitored. *T. gondii* tachyzoites were expected to colonize the brain tissue and form cysts after 30 dpi. The mice were euthanized at this stage, and brain tissues were dissected and homogenized. The number of cysts was counted under a microscope for subsequent oral infection of C57BL/6J mice. The C57BL/6J mice were randomly divided into the blank control group (CJN, *n* = 10) and the *T. gondii* infected group (CJI, *n* = 50). All the animals were randomly grouped, labeled, and acclimatized for ten days before the experiment. Each mouse in the CJN group received an oral gavage of 200 µL of phosphate-buffered saline (PBS). In contrast, each CJI mouse was gavaged with 100 *T. gondii* cysts in a total volume of 100 µL. The grouping protocols, gavage infection, and sample collection are presented in Figure 1A.

### 2.3. Clinical Observation and Sample Collection of Mice

The mice’s diet, drinking habits, and activity levels were continuously monitored before and throughout the 60-day experiment. Body weight was measured using an electronic scale. The rectal temperature was recorded with a probe thermometer every three days. Commercial nutritional jelly (Ready Jelly^®^ Recovery, Shenzhen, Chian) was administered promptly when the weight loss was greater than 15%. Weight loss exceeding 25% was established as the humane endpoint for euthanasia to ensure animal welfare. Mice were euthanized using a carbon dioxide instrument (30–70% concentrations). A clinical examination was conducted on the dead body and fur surface after euthanasia. The animals’ abdominal and thoracic cavities were dissected macroscopically, focusing on the intestines, spleen, liver, lungs, and heart. The samples included whole blood, ileum, colon, heart, liver, spleen, lungs, kidneys, brain, eyes, thymus, and hind leg muscle. Mouse sera and tissues (including the brain) were collected for immunological and histopathological analyses under specified conditions. Infected mice were monitored for 60 consecutive dpi. During monitoring, tissue sampling and analysis were performed at various intervals to assess infection progression and immune responses.

### 2.4. Detection of T. gondii with Real-Time q-PCR

Qualitative and quantitative analyses of the invasion, dissemination, proliferation, and colonization of *T. gondii* tachyzoites and cysts in different mouse tissues and organs were qualitatively and quantitatively analyzed using qPCR techniques. DNA was extracted from the tissue homogenate, and *Toxoplasma* nucleic acid was detected by targeting the *T. gondii* B1 gene using qPCR [29]. A TaqMan qPCR system targeting the *T. gondii* B1 locus (GenBank: AF179871) was applied with forward (TOXO-F: 5′-TCCCCTCTGCTGGCGAAAAGT-3′) and reverse (TOXO-R: 5′-AGCGTTCGTGGTCAACTATCGATTG-3′) primers paired with a dual-labeled (6FAM-TAMRA) hydrolysis probe. Genomic DNA was isolated from tissue homogenates using the Magnetic bead-based nucleic acid extraction kit (Sbeadex livestock kit, LGC, London, England) according to the manufacturer’s protocol. Amplification reactions (25 μL) comprising 12.5 μL of 2 × Premix Ex Taq (Probe qPCR) (TaKaRa, Dalian, China), 0.5 μL of each primer, 1 μL of probe, and 2 μL of template DNA were processed on a real-time PCR System (BioRad CFX96, Hercules, CA, USA). The thermal cycling parameters were set as follows: initial denaturation at 95 °C for 10 min, followed by 40 cycles of 95 °C for 15 s and 60 °C for 1 min. Real-time fluorescence monitoring enabled the automated determination of cycle threshold (Ct) values. C57BL/6J mice were orally inoculated with *Toxoplasma* cysts, with their tissues and organs being collected at specified periods post-infection (1–10 days, 15 days, 20 days, 30 days, 45 days, and 60 days). We analyzed the dynamics of *T. gondii* invasion, dissemination, proliferation, and colonization across different tissues and organs at each time point using the amplified CT values and the reference curve.

### 2.5. Serum T. gondii-Specific Antibodies

Serum was collected from mice at various durations following *T. gondii* infection to assess the levels of *T. gondii*-specific antibodies, specifically IgG. This measurement was performed using a commercially available ELISA kit (VRL Asia, Suzhou, China), following the manufacturer’s instructions. Each experiment included a positive control and a negative control to ensure validity. Specific criteria for a valid experimental design included an optical density (OD) of the negative control < 0.25 and a positive control OD ≥ 0.60. Invalid experiments were repeated until the defined criteria were met. The sample was considered positive when its OD value was ≥0.5 and negative when the corresponding OD was <0.5.

### 2.6. Histopathological Analysis

Brain tissue samples were collected from mice in the CJN and CJI groups 30 dpi and fixed in 4% (*v*/*v*) neutral-buffered formalin. These tissues were processed using standard histological techniques and embedded in paraffin to analyze the pathological lesions and distribution of *T. gondii* antigens. The paraffin sections were cut into 5-µm thick slices and stained with hematoxylin and eosin (H&E). Histopathological changes were evaluated using light microscopy (Olympus VS200, Tokyo, Japan). The methodology for pathological analysis was based on established protocols from previous research [30].

### 2.7. Immunofluorescence

Paraffin sections from the CJN and CJI mice were deparaffinized in water. The paraffin-free sections were subjected to antigen retrieval using an EDTA buffer (pH 8.0). These tissue sections were blocked with 10% bovine serum albumin at 37 °C for 30 min, and then the blocking buffer was removed. The resulting sections were incubated at 4 °C overnight with primary antibodies, specifically goat anti-*T. gondii* polyclonal antibody (1:1000, Invitrogen, Carlsbad, CA, USA). The incubated sections were washed three times with PBS and further incubated with secondary antibodies (1:500, Servicebio, Wuhan, China) at room temperature for 50 min. The 4′,6-diamidino-2-phenylindole was added and incubated at room temperature in the dark for 10 min to counterstain the cell nuclei. Finally, the sections were sealed with fluorescent mounting media for further analysis [31]. The stained sections were examined under a fluorescence microscope (Olympus VS200, Japan).

### 2.8. Statistical Analysis

GraphPad Prism 8.0.2 (GraphPad Software Inc., San Diego, CA, USA) was employed for analyses and graph creation. All the experiments were conducted at least three times, and the results are presented as the mean ± standard error (SEM). Body weight changes were determined based on two-way ANOVA. Cumulative mortality was plotted using Kaplan–Meier survival curves and analyzed with the Log-rank (Mantel–Cox) test. Differences between groups were assessed using Student’s *t*-test or the Mann–Whitney test, as indicated in figure legends. *p* < 0.05 was considered statistically significant. * *p* < 0.05, ** *p* < 0.01, *** *p* < 0.001, and **** *p* < 0.0001 indicate a statistically significant difference, while ‘ns’ represents no significant differences.

## 3. Results

### 3.1. T. gondii-Infected Mouse Model and Analysis of Clinical Status

The cultivation of rapidly replicating *T. gondii*, the cyst preparation, and the experimental design for C57BL/6J mice are illustrated in Figure 1A, B. Approximately after 5 dpi with *T. gondii* cysts, notable changes in mice were observed, including reduced activity, rough fur, and a tendency to huddle in cage corners (Figure 1C). Daily clinical observations revealed a marked decrease in appetite and water intake at 3 dpi, accompanied by fever or an elevated body temperature, indicating the onset of the acute infection phase. The mice underwent an evident decline in body weight as of 5 dpi. In addition, the average body temperature decreased after peaking above 37 °C.

Mortality occurred roughly one week post-infection. The minimum body weight and temperature were observed by 10 dpi, leading to significant mortality rates (Figure 1D–F). The survivors transitioned from the acute phase to the chronic phase between ten and 15 dpi. Their conditions had improved by approximately two weeks post-infection. This transition was characterized by slight weight gain despite persistently lower body temperatures than normal levels.

The body weight of mice in the CJI group slowly increased and stabilized within 30–60 dpi. Despite this improvement, the temperature remained significantly lower than in the CJN group. Although the body temperature returned to normal levels, occasional deaths were still recorded among the infected mice (Figure 1D–F).

### 3.2. Oral Infection of Mice with T. gondii Cysts Induces Inflammatory Bowel Disease and Multi-Organ System Damage

**Figure 2:**
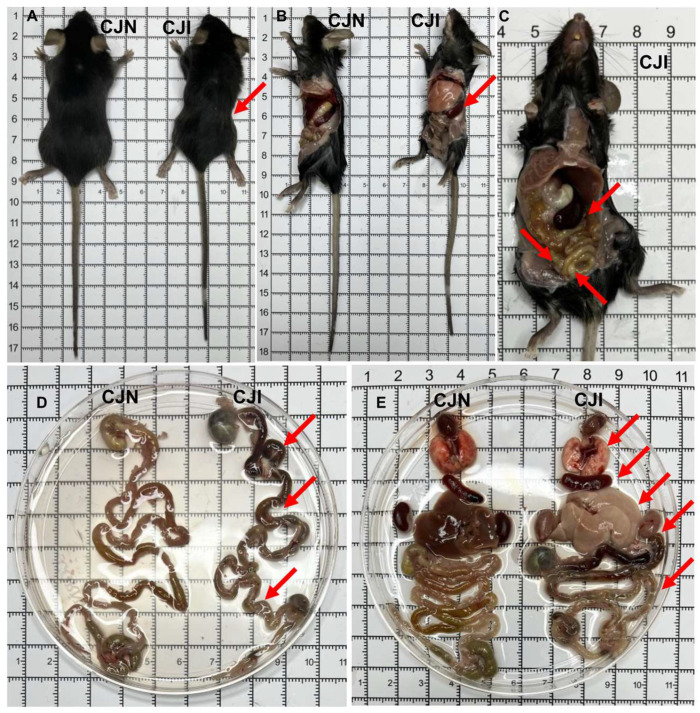
Oral infection of C57BL/6J mice with *T. gondii* cysts leads to multi-organ inflammatory damage. (**A**) Representative images of the torso from the CJN and CJI groups. (**B**,**C**) Gross observations of the peritoneal cavity in the CJN and CJI groups. (**D**,**E**) Anatomical comparisons of various organ systems, including the digestive system (small intestine, large intestine, and liver), circulatory and respiratory systems (heart, lungs, and kidneys), and immune system (spleen) between the CJN and CJI groups. The red arrows indicate organs and tissues that have suffered from severe pathological changes and injuries.

Acutely infected mice that reached humane endpoints were immediately euthanized, followed by gross anatomical and pathological examination. Infected mice exhibited noticeable signs of emaciation and rough fur (Figure 2A). Gross anatomical analysis revealed significant splenomegaly in the abdominal cavity, accompanied by enlarged intestinal lymph nodes (Figure 2B,C). The surface areas of the jejunum and ileum displayed black discoloration during gastrointestinal tract dissection, indicating severe hemorrhage, tissue damage, and necrosis. Additionally, the cecum and colon were markedly reduced and shortened. Extensive damage and necrosis were observed throughout the intestinal tissue, leading to lethal enteritis (Figure 2D).

Comparative dissection further revealed that the hearts of *T. gondii*-infected mice in the acute infection phase were slightly shrunken, while their spleens were markedly enlarged. The lungs, liver, and kidneys exhibited a pale appearance, and petechial hemorrhages were observed on the surfaces of the lungs and kidneys (Figure 2E). Based on the above observations, *T. gondii* infection led to significant pathological damage and inflammation across multiple organs and systems in mice, resulting in multi-organ failure and death.

### 3.3. T. gondii Loads Vary Across Multiple Time Points and Various Organs

As mentioned, brain tissues of mice infected with *T. gondii* were homogenized, and qPCR analysis targeting the *Toxoplasma* B1 gene demonstrated an inverse relationship between dilution concentration and CT values. This analysis confirmed our method’s effectiveness in detecting the presence of *T. gondii* cysts. The qPCR method facilitates the qualitative and quantitative analysis of *T. gondii* load in infected mouse tissues with high specificity and sensitivity, laying a groundwork for follow-up detection applications.

**Figure 3:**
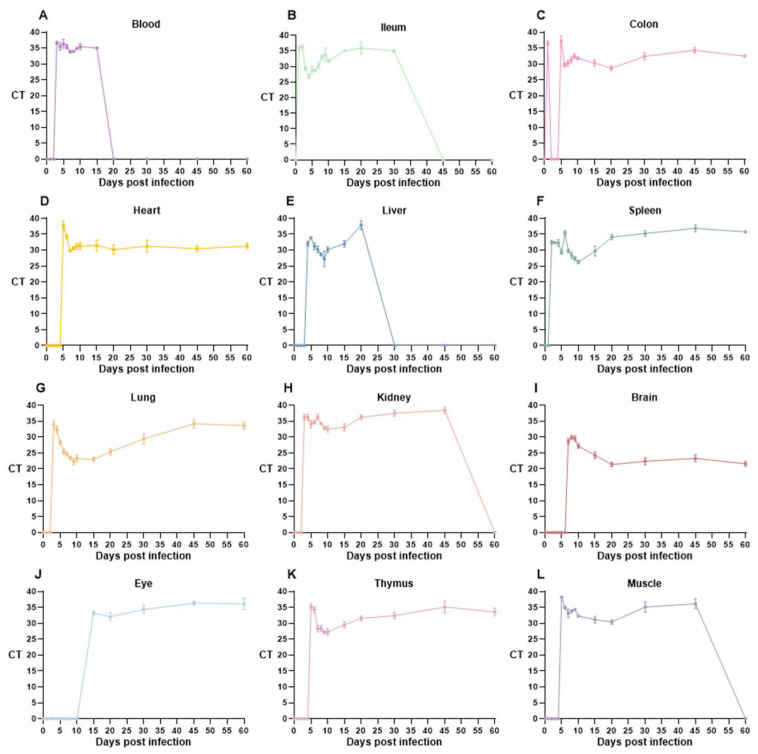
Time-course analysis of CT value changes targeting the *Toxoplasma* B1 gene via qPCR. For mice orally infected at 1–10 days, 15 days, 20 days, 30 days, 45 days, and 60 days, the blood, ileum, colon, heart, liver, spleen, lung, kidney, brain, eye, thymus, and muscle were collected for nucleic acid extraction. Furthermore, the *T. gondii* B1 gene was detected using qPCR. Tissues including blood, ileum, colon, heart, liver, spleen, lung, kidney, brain, eye, thymus, and muscle were tested, as shown in panels (**A**–**L**).

Subsequently, we evaluated changes in CT values and tissue samples (blood, duodenum, ileum, heart, liver, spleen, lungs, kidneys, brain, eyes, thymus, muscle) across 15 intervals (1–10, 15, 20, 30, 45, and 60 dpi) using the qualitative and quantitative qPCR method targeting the *Toxoplasma* B1 gene (Figure 3). Qualitative analysis was initially performed using CT values, followed by quantitative analysis. The detection result was considered positive when 18 ≤ CT ≤ 38, with a lower CT value indicating a higher *T. gondii* load. Infection intensity is denoted as a ‘+’ symbol based on CT levels. The dynamic distribution of *T. gondii* across various tissues over time is detailed in Table 1.

Oral gavage of *T. gondii* cysts triggered the infection. The introduced cysts were broken down by gastric acid in the host stomach, releasing parasites into the gastrointestinal environment. During the acute phase of infection (1–2 dpi), parasites migrated to the duodenum and colonic tissues and rapidly spread to the spleen via the bloodstream. The proliferating *T. gondii* forms disseminated to the lungs, kidneys, and liver through the bloodstream between 3 and 4 dpi. We detected the presence of *T. gondii* in the heart, thymus, and leg muscles at 5 dpi. By 7 dpi, parasites breached the blood–brain barrier (BBB) and reached the brain tissues. *T. gondii* rapidly disseminated to most organs and tissues within one week following infection. The lungs of acutely infected mice exhibited significant parasite proliferation (Figure 3G and Table 1). The infection subsequently progressed into the chronic phase. *T. gondii* breached the blood–retinal barrier and invaded the eye tissues at 15 dpi. This parasite progressively colonized the brain and proliferated considerably (Figure 3I and Table 1). Key tissues, including the heart, brain, and leg muscles, shifted from rapidly proliferating forms (tachyzoites) to bradyzoites, initiating a dormant state as cysts. These cysts remain isolated from the host’s immune system and can persist for the host’s lifetime, facilitating transmission to intermediate and definitive hosts.

### 3.4. Changes in Toxoplasma-Specific Antibodies in Mouse Serum

**Figure 4:**
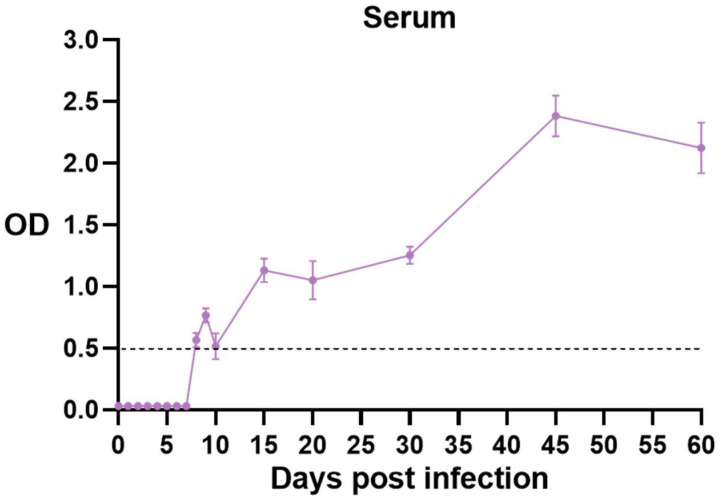
Changes in *T. gondii*-specific IgG antibody titers in mouse serum. After oral infection of mice with *T. gondii*, mouse serum was collected at 1–10 days, 15 days, 20 days, 30 days, 45 days, and 60 days, respectively, and *T. gondii* IgG antibodies were detected using ELISA. *Toxoplasma* antibodies were first detected on 8th dpi. The antibody titer levels continued to rise, peaked at 45 dpi, then gradually declined, and stabilized until 60 dpi.

Serum samples were collected at various dpi intervals, and *T. gondii*-specific IgG antibodies were quantified using an ELISA kit. The results revealed progressive changes in antibody titers (Figure 4). *T. gondii* IgG antibodies were detectable in C57BL/6J mice by the 8th dpi. The OD value was nearly doubled by 15 dpi, indicating a consistent increase. This value peaked at 45 dpi, remained elevated within 60 dpi, then slightly diminished while remaining at a relatively high level. These findings demonstrated that *T. gondii* infection rapidly triggered IgG antibody production during the acute phase. In addition, the concentration of these antibodies increased when the infection shifted into the chronic phase and further elevated for more than two months. The detection method employed here can identify serum antibody positivity approximately one week into the acute phase of infection, showcasing good specificity and high sensitivity. These results underpin the development of immune serological testing technologies for *T. gondii* infections.

### 3.5. Histological and Immunofluorescence Analysis of Mouse Brain

**Figure 5:**
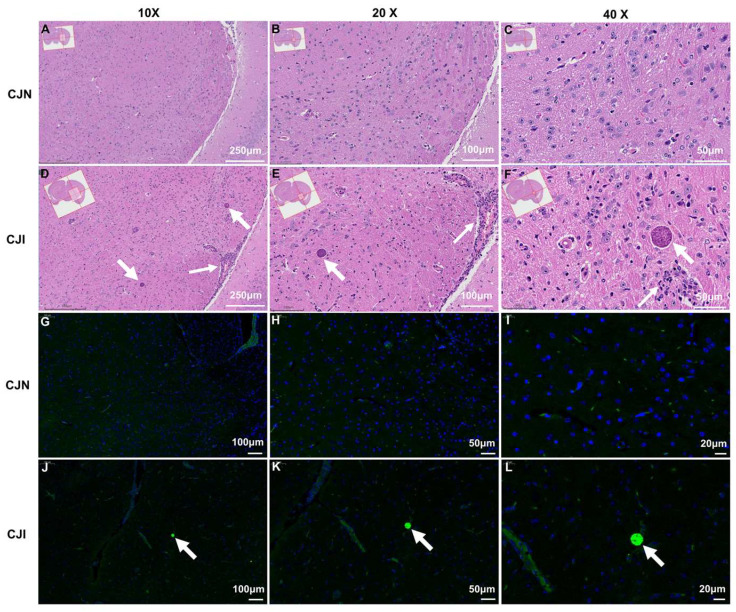
H&E staining and immunofluorescence analysis within 30 days after *T. gondii* infection. (**A**–**C**) H&E staining of the mouse brain tissue samples from the CJN group (scale bars = 250 μm, ×10 objective; scale bars = 100 μm, ×20 objective; scale bars = 20 μm, ×40 objective). Only representative images are presented. (**D**–**F**) H&E staining of the mouse brain tissue samples from the CJI group (scale bars = 250 μm, ×10 objective; scale bars = 100 μm, ×20 objective; scale bars = 50 μm, ×40 objective). Only representative images are presented. Focal mononuclear cells infiltrating into the brain and meningitis with infiltrating mononuclear cells (indicated by thin arrow); Vascular cuffing with infiltrating mononuclear cells (thin arrow); *T. gondii* cyst (thick arrow). (**G**–**I**) Immunofluorescence analysis of the mouse brain tissue samples from the CJN group (scale bars = 100 μm, ×10 objective; scale bars = 50 μm, ×20 objective; scale bars = 20 μm, ×40 objective). Only representative images are presented. (**J**–**L**) Immunofluorescence analysis of the mouse brain tissue samples from the CJI group (scale bars = 100 μm, ×10 objective; scale bars = 50 μm, ×20 objective; scale bars = 20 μm, ×40 objective). Only representative images are presented. *T. gondii* cyst (thick arrow).

Mice from the CJN and CJI groups were humanely euthanized at 30 dpi, and their brain tissues were harvested for histological examination using H&E staining and immunofluorescence analysis. *T. gondii* breached the BBB, infiltrated, and proliferated within the brain tissue by 7 dpi. Pathological examination revealed that *T. gondii* invasion led to significant monocyte infiltration, inducing meningitis (Figure 5A–F). This parasite transitioned from the rapidly replicating tachyzoites to the slowly growing bradyzoites when the infection progressed from the acute to the chronic phase, forming cysts (Figure 5F). Immunofluorescence analysis using recombinant *T. gondii* proteins effectively detected the presence of cysts within the hippocampal region of mouse brain tissue (Figure 5J–L). This method facilitates qualitative and quantitative assessments of parasite loads in brain tissue and supports research on the three-dimensional distribution of *T. gondii* cysts within the host brain.

## 4. Discussion

**Figure 6:**
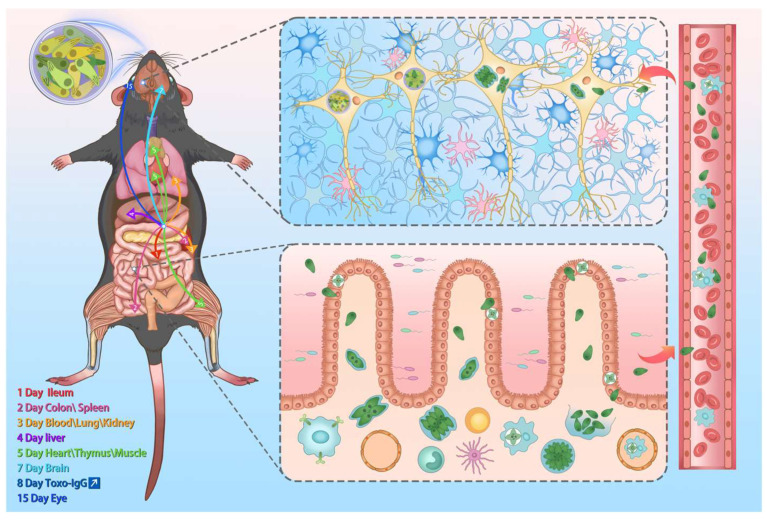
A diagram of *T. gondii* invasion and colonization in C57BL/6J mice at spatiotemporal points. *T. gondii* cysts ingested orally are digested in the stomach, where stomach acid stimulates their breakdown, releasing numerous sporozoites. These sporozoites invade duodenal epithelial cells on the first day of infection and spread to the colon and spleen on the second day, causing inflammatory enteritis. By the third day, *T. gondii* enters the bloodstream and spreads to the lungs and kidneys. On the fourth day, it further disseminates to the liver and reaches the heart, thymus, and muscular tissue by the fifth day. On the seventh day of infection, *T. gondii* crosses the BBB to infect the brain. On the eighth day, specific IgG antibodies are first detected in the serum of mice, with levels rising and remaining elevated for over two months. *T. gondii* crosses the blood–ocular barrier and infects the eyes by the fifteenth day. During the third week post-infection, *T. gondii* forms cysts in tissues such as the brain and muscles. At 30 dpi, histological sections and immunofluorescence assays reveal that cysts, preferentially residing in the brain, have formed in the tissue.

*T. gondii* is an important zoonotic parasite that can infect virtually all nucleated cells in warm-blooded animals, primarily transmitted through contaminated food, soil, and direct contact [1]. However, systematic research on the invasion, dissemination, proliferation, and colonization dynamics of orally ingested *T. gondii* cysts in mice by integrating clinical diagnostics, molecular biology, histopathology, and immunology remains limited [32,33]. Despite measurable humoral immunity in murine *Toxoplasma* models, therapeutic progress is hindered by unresolved mechanisms regulating tachyzoite–bradyzoite conversion, seroconversion thresholds, IgG neutralization kinetics, and immune memory durability. These critical gaps present challenges in elucidating immune–parasitic oscillatory dynamics that govern chronic, persistent infection [34,35]. Humans are typically infected with *T. gondii* by consuming undercooked meat contaminated with cysts, particularly pork and lamb [36,37]. Thus, clarifying the transmission pathways of food-borne cysts is essential for developing effective prevention, control, and treatment strategies [38]. Accordingly, we constructed a C57BL/6J mouse model, a strain susceptible to *T. gondii*, to investigate the spatiotemporal diffusion and colonization of *T. gondii* cysts following oral infection and the antibody responses (Figure 6).

During acute *T. gondii* infection, C57BL/6J mice may manifest various clinical signs, including respiratory distress, multi-organ failure, and death [24,39]. Our findings are consistent with previous results showing that *T. gondii* infection can induce lethal enteritis and abnormal proliferation in intestinal lymph nodes and the spleen [24,40,41]. Oral infection often triggers a “cytokine storm” in the small intestine, driven by Th1-type cytokines. Notably, IFN-γ mediates Paneth cell death by inhibiting mTOR [42], a mechanism resembling that in human inflammatory bowel diseases such as Crohn’s disease. Thus, oral *T. gondii* infection can cause immunopathology in various animals, in addition to C57BL/6J mice [38]. Chronic *T. gondii* infection is also associated with increased susceptibility to colitis [40].

Susceptibility to *T. gondii* varies among mouse strains and is greater in C57BL/6 mice than BALB/c mice [22]. IL-10 plays a key role in reducing mortality and preventing intestinal necrosis in BALB/c and C57BL/6 mice after oral infection [43]. Additionally, oral *T. gondii* infection has been demonstrated to cause gut microbiota dysbiosis, impair intestinal barrier integrity, and induce colonic inflammation [39,44]. Gram-negative bacteria can exacerbate small intestinal Th1-type immunopathology [45]. However, interventions such as fecal microbiota transplant using probiotics (e.g., *Lactobacillus* and *Bacteroides*) have proved to alleviate intestinal inflammation in infected mice [46].

This study showed that *T. gondii* preferentially targeted the lung tissue during acute infection, thereby disseminating through the bloodstream and causing severe pneumonia. These results align with previous research reporting that a *T. gondii* pneumonia model exhibited weight loss, ruffled fur, and respiratory rales, complicated by severe lymphocytic infiltration and pulmonary edema [47]. The lung’s role in pulmonary and systemic circulation facilitates efficient *T. gondii* dissemination, making it a preferred site for infection. This pattern may provide insights into the higher seropositivity rate (60.94%) in lung cancer patients than the general population [48].

This study demonstrated that *T. gondii* crossed the BBB and colonized the brain approximately 7 dpi. This finding corroborates previous work showing that *T. gondii* invaded vascular endothelial cells, proliferated, and crossed the BBB into the central nervous system (CNS) [49]. Researchers reported increased inflammatory monocytes in the blood 4 dpi [50]. *T. gondii* was detected in brain tissues by 7 dpi, indicating its successful passage through the BBB, possibly even earlier than this period [51].

Mice enter the chronic infection stage by 14 dpi. In this case, the animals show partial improvements in locomotor activity, feeding patterns, and body mass indices. However, these parameters remain subnormal compared to uninfected controls throughout persistent infection. *T. gondii* persists as bradyzoites in low-immunity tissues such as the brain, skeletal muscles, eyes, and heart, leading to lifelong chronic infection [52]. It crosses the BBB through various pathways, including paracellular [53], transcellular [49], and the “Trojan horse” mechanism [50]. The resulting CNS damage can trigger neuropsychiatric symptoms [54]. The reason is that *T. gondii* induces inflammation in various CNS cells and stimulates the production of inflammatory cytokines and chemokines [55,56].

This study first detected *T. gondii*-specific IgG antibodies in mouse serum at 8 dpi, with levels gradually increasing and remaining elevated for two months. During chronic infection, its transformation from tachyzoites to bradyzoites facilitated immune detection evasion by forming a highly glycosylated cyst wall [57]. Chronic infection may manifest no overt symptoms under normal immune function; in contrast, declined host immunity can trigger cyst activation, leading to *Toxoplasma* encephalitis [58,59]. This reactivation is linked to various neurological symptoms, including cognitive impairments [60], suicide [61], and schizophrenia [62]. Researchers demonstrated that chronic *T. gondii* infection could induce anxiety-like behavior in mice via the “gut–brain axis”, possibly modulated through gut microbiota changes [63].

Initial infection triggers an innate immune response, recruiting monocytes from the bone marrow to the site of infection. These monocytes release cytokines and chemokines, differentiating into macrophages and dendritic cells, contributing to infection control [64]. GTPases induced by IFN-γ, such as IRG and GBP [65,66], are critical for host resistance [32]. Both the current and previous studies highlight that the brain is a primary target for *T. gondii* infection in mammals, with neurons being the preferred target of colonization and proliferation [67,68,69,70].

## 5. Conclusions

This research reveals that *T. gondii* cysts ingested by susceptible C57BL/6J mice are broken down by stomach acid, facilitating the rapid spread of sporozoites to the gastrointestinal tract and spleen. *T. gondii* enters the bloodstream within 3 dpi, causing fever, weight loss, and even fatal enteritis. *T. gondii* infiltrates most tissues, crosses the BBB, and colonizes the brain within 7 dpi, inducing inflammation, tissue damage, and an antibody response that persists for months. The lungs are the primary target during acute infection, and the brain is a predilection site in the chronic infection stage. The infection transitions to the chronic phase two weeks post-infection, with the parasite transforming into bradyzoites. These bradyzoites trigger lifelong inflammation and form cysts that persist within the host. These findings provide valuable insights into the underlying mechanisms of *T. gondii* pathogenesis and host–*T. gondii* interaction.

## Figures and Tables

**Table 1:** *T. gondii* load in various tissues and organs of the infected mice at different time points detected using qPCR targeting the B1 gene.

Sample	CT Value Classification with Days Post Infection														
	1	2	3	4	5	6	7	8	9	10	15	20	30	45	60
Blood	−	−	±	+	+	+	++	++	++	+	+	−	−	−	−
Ileum	+	+	+++	+++	+++	+++	+++	++	++	++	+	+	+	−	−
Colon	−	+	−	−	+	++	++	++	++	++	++	+++	++	+	++
Heart	−	−	−	−	+	+	+++	++	++	++	++	++	++	++	++
Liver	−	−	−	++	++	++	++	+++	+++	++	++	+	−	−	−
Spleen	−	++	++	++	+++	+	+++	+++	+++	+++	+++	++	+	+	+
Lung	−	−	++	++	+++	+++	+++	++++	++++	++++	++++	++++	+++	++	+
Kidney	−	−	+	+	++	+	+	+	++	++	++	+	+	±	−
Brain	−	−	−	−	−	−	+++	+++	+++	+++	++++	+++++	++++	++++	+++++
Eye	−	−	−	−	−	−	−	−	−	−	++	++	+	+	+
Thymus	−	−	−	−	+	+	+++	+++	+++	+++	+++	++	++	+	++
Muscle	−	−	−	−	±	+	++	++	++	++	++	++	+	+	−

CT is not available (N/A) and marked as “−”; 18 ≤ CT ≤ 38 is classified as positive (POS); among them, 34 ≤ CT ≤ 38 is marked as “+”, 30 ≤ CT < 34 is denoted as “++”, 26 ≤ CT < 30 is represented by “+++”, 22 ≤ CT < 26 corresponds to “++++”, and 18 ≤ CT < 22 is described as “+++++”; 38 < CT ≤ 40 is classified as indeterminate (IND) and marked as “±”. The yellow background marking indicates the initial detection of *T. gondii* in the infected organ.

## Data Availability

The datasets supporting the findings of this article are included within the paper.
